# POEMS syndrome: clinical update

**DOI:** 10.1007/s00415-018-9110-6

**Published:** 2018-11-29

**Authors:** Rachel Brown, Lionel Ginsberg

**Affiliations:** 1 Department of Neurology, Royal Free Hospital, Pond Street, London, NW3 2QG UK; 2 Queen Square Institute of Neurology, University College London, London, UK

**Keywords:** Neuropathy, Paraproteinaemia, Monoclonal gammopathy, Vascular endothelial growth factor (VEGF)

## Abstract

POEMS syndrome (polyneuropathy, organomegaly, endocrinopathy, M-protein, skin changes) is a rare paraneoplastic syndrome, caused by a plasma cell proliferative disorder, which is most commonly lambda restricted. The neurological hallmark, which forms one of the mandatory criteria for diagnosis, is a subacute onset demyelinating neuropathy, which can be rapidly disabling and painful. A number of multi-system features are also characteristic of this disorder, and certainly not restricted to those included in its acronym, which though limited, remains a useful and memorable name, helping distinguish POEMS syndrome from other paraproteinaemic neuropathies. The discovery of vascular endothelial growth factor (VEGF) in association with POEMS syndrome has been extremely useful in aiding clinical diagnosis, and monitoring response to treatment, as well as helping understand the underlying mechanism of disease. Interestingly, however, treatment targeting VEGF has been disappointing, suggesting other disease mechanisms or inflammatory processes are also important. Current understanding of the pathogenesis of POEMS syndrome is outlined in detail in the accompanying article by Cerri et al. Here, we review the clinical features of POEMS syndrome, differential diagnosis and available treatment options, based on current literature.

## Introduction

The diagnosis of POEMS syndrome (polyneuropathy, organomegaly, endocrinopathy, M-protein, skin changes), based on the current Dispenzieri diagnostic criteria [1], requires the presence of both mandatory criteria (a polyneuropathy and a monoclonal plasma cell-proliferative disorder, almost always lambda restricted), and at least one major and one minor criterion (Table 1). POEMS syndrome differs from other paraproteinaemic and inflammatory neuropathies by its multi-organ involvement, thought to be caused by elevated pro-inflammatory and angiogenic cytokines. Multi-organ features extend beyond those included in its acronym, and not all features included in the acronym are required for diagnosis.

**Table 1:** Diagnostic criteria

Mandatory criteria
Polyneuropathy
Monoclonal plasmaproliferative disorder
Other major criteria
Sclerotic bone lesions
Castleman’s disease
Elevated VEGF
Minor criteria
Organomegaly
Oedema
Endocrinopathy
Skin changes
Papilloedema
Thrombocytosis/Polycythaemia
Other symptoms and signs
Clubbing
Weight loss
Hyperhidrosis
Pulmonary hypertension
Restrictive lung disease
Thrombotic diathesis
Low vitamin B12 level
Diarrhoea

POEMS syndrome has a median age of onset in the sixth decade and a slight male preponderance [2]. Patients may present to one of a number of specialty clinics depending upon the initial symptoms. In the neurology clinic, patients typically describe a subacute, painful, distal neuropathy. If POEMS syndrome is suspected, a thorough systemic examination and timely organisation of relevant investigations are required to elicit all features that might aid diagnosis.

POEMS syndrome remains a rare disease and evidence for treatment is largely limited to retrospective cohort studies or case reports. Current treatment strategies all target the underlying plasma cell clone, with the exception of bevacizumab, a monoclonal antibody targeting vascular endothelial growth factor (VEGF), which has had disappointing results. Management can be complicated, and POEMS syndrome can be fatal. With the right treatment, however, prognosis in many patients can be very good. Joint specialty clinics, usually staffed by haematologists and neurologists, can be valuable.

## Clinical features

### Polyneuropathy

#### Clinical findings

Patients typically present with a subacute, distal, symmetrical, sensorimotor neuropathy, frequently painful, with allodynia and hyperpathia [3, 4]. Neuropathy is a common first clinical feature, and may be the only feature at first presentation [3, 5].

The lower limbs are affected earlier, and more severely, than the upper limbs [3, 6, 7]. Sensory symptoms usually precede motor symptoms [6]. Many patients quickly become wheelchair- or bed-bound due to weakness or pain. Clinical examination may reveal distal wasting, weakness and sensory loss affecting both large and small fibre sensory modalities [3].

#### Neurophysiology

Electrodiagnostic studies demonstrate a length-dependent sensorimotor neuropathy, typically demyelinating, but with axonal degeneration [5, 7]. Conduction block is not usually present [5, 7]. In motor studies, reduction in motor conduction velocity (MCV) is an early sign, however, patients often already have significant axonal loss at presentation [7]. Sensory studies show reduction of, or often absent, sensory nerve action potentials [3, 5].

POEMS syndrome is distinguishable from other polyneuropathies on electrodiagnostic studies, with some overlap. In a study of 51 patients with POEMS syndrome, 70% met the European Federation of Neurological Societies and Peripheral Nerve Society criteria for definite chronic inflammatory demyelinating polyradiculoneuropathy (CIDP) [3]. Reduction in MCV is typically seen in similar proportions of patients with POEMS syndrome, CIDP and CMT1a [3, 8]. However, while distal motor latencies tend to be prolonged in POEMS syndrome, they are less prolonged (and less often) than in CIDP or CMT1a [3, 8]. This is thought to indicate that slowing in POEMS syndrome is more prominent in intermediate than distal segments, suggesting a potentially different disease pathogenesis from other inflammatory neuropathies [3, 5, 8, 9]. Conduction block is much more common in CIDP than POEMS syndrome [3, 8], and the discrepancy in severity between upper and lower limb axonal loss is more pronounced in POEMS syndrome [3, 8].

#### Neuropathology

Nerve biopsy can be used to support a diagnosis of POEMS syndrome, though in practice it is not essential, especially when other clinical and paraclinical findings already fulfil the diagnostic criteria. A number of pathological hallmarks for POEMS syndrome have been identified and are outlined in detail in the accompanying article by Cerri et al. Of all these features, the finding of regular uncompacted myelin lamellae (UML) in ≥ 1% of myelinated nerve fibres on electron microscopy, is thought to be highly specific for POEMS syndrome, though not 100% pathognomonic [10, 11].

### Plasma cell dyscrasia

The plasma cell disorder underlying POEMS syndrome is typically IgA or IgG lambda restricted [12]. A paraprotein can be identified on serum protein electrophoresis and/or immunofixation in most patients, though in our own experience, and as noted in larger series [6, 12], some patients do not have a detectable paraprotein, even after serial testing. Demonstrating a plasma cell clone in these patients requires more extensive investigation.

#### Bone marrow biopsy

Patients with POEMS syndrome typically have few monoclonal plasma cells on iliac crest biopsies. In patients with localised disease, iliac crest biopsies can be normal [6, 12]. In a study of 87 patients at the Mayo clinic, the median percentage of plasma cells in 67 pre-treatment bone marrow specimens was < 5%, with monoclonal plasma cells detected in two-thirds [12]. A typical finding in around half of pre-treatment specimens was the presence of histologically reactive-appearing lymphoid aggregates containing mixed B- and T-cells, with a plasma cell rim, most commonly lambda restricted (though some polytypic), thought to be unique to POEMS syndrome. Other frequent findings included megakaryocyte hyperplasia and clustering, and atypical megakaryocyte appearances. Peripheral blood smears were normal.

Within osteosclerotic lesions, atypical plasma cells infiltrate normal marrow with sclerosis of the bony lamellae [6]. Bone marrow aspirates from osteosclerotic lesions in two patients, by Kulkarni et al., revealed high levels of plasmacytosis (> 10%), while more sclerotic lesions were hypo- or acellular [6].

### Other major criteria

#### Castleman’s disease

Castleman’s disease (CD) is a rare heterogeneous lymph node disorder associated with high levels of interleukin-6 (IL-6) [13]. Patients with CD can have neuropathy, with or without POEMS syndrome. In a minority, CD co-exists with neuropathy and a POEMS-like syndrome, without evidence of a monoclonal plasma cell disorder. These patients are excluded in the current diagnostic criteria for POEMS syndrome as they do not fulfil the mandatory criteria, and are felt to have a different disease/pathogenesis [1]. The type and severity of neuropathy differs depending upon the presence or absence of POEMS syndrome or CD. Patients with CD and neuropathy without POEMS syndrome typically have a mild, painless, distal sensory neuropathy, while patients with POEMS syndrome +/- CD typically have a painful sensorimotor neuropathy, most severe in those without CD [14].

In a study of 113 patients with biopsy confirmed CD treated between 1948 and 2002, 18% met criteria for POEMS syndrome [15]. Conversely, in a study of 87 patients with POEMS syndrome, 10 had CD and 5 had CD-like features on lymph node biopsy [12]. Patients with POEMS syndrome mostly exhibit HHV8 negative multicentric CD (MCD) [12, 14, 15].

Overall survival is dependent on the presence or absence of CD, osteosclerotic lesions, and POEMS syndrome. In a Mayo clinic cohort, 5-year survival was very good in patients with unicentric CD (91%) or with MCD with osteosclerotic lesions and POEMS syndrome (90%), good in patients with MCD without POEMS syndrome (65%), and very poor in patients with MCD and POEMS syndrome without osteosclerotic bone lesions (27%) [15].

#### Sclerotic bone lesions

POEMS syndrome is associated with predominantly osteosclerotic bone lesions, which can have mixed sclerotic-lytic components, and are also referred to as osteosclerotic myeloma. Lesions are often small, and multiple; in a study of 28 patients undergoing chest and abdominal CT imaging, 68% of patients had multiple bone lesions, with 146 lesions identified in total, 97% reported as sclerotic, and 71% <10 mm in diameter [16]. Further imaging by ^99m^Tc-HMDP bone scintigraphy enabled the pick-up of additional lesions in the skull and long bones, not in the field of view of CT, demonstrating a benefit from imaging the complete skeleton, and of using dual imaging modalities. ^18^F-FDG PET/CT imaging can also aid diagnosis and help identify lesions suitable for biopsy [17]. Lesions are commonly found in the pelvis, thoracic and lumbar vertebrae, and ribs, and also occur in the scapula, clavicle, sternum, skull and long bones [16, 17].

#### Vascular endothelial growth factor

Elevated VEGF is highly specific for POEMS syndrome (though not pathognomonic) and thought to be involved in the pathophysiology of systemic features including organomegaly and volume overload. Serum VEGF levels reflect disease activity, falling with treatment and rising with disease progression or relapse. Monitoring VEGF levels may be useful as a disease prognostic marker; Misawa et al. showed that reduction in serum VEGF levels to within the normal range by 6-months post-treatment was associated with longer relapse-free survival and better outcomes [18].

### Minor criteria

#### Organomegaly

Clinical and radiological studies have shown that imaging, particularly CT, has higher sensitivity in identifying organomegaly compared to clinical examination [6, 19]. In a retrospective cohort of 29 patients diagnosed with POEMS syndrome in India between 1983 and 2009, all had organomegaly, including hepatomegaly (28/29), splenomegaly (21/29) and lymphadenopathy (7/29) [6]. The reporting of organomegaly is variable, however, and is lower in other cohorts [2].

#### Extravascular volume overload

Fluid overload may present peripherally or centrally. In a study of 91 patients undergoing ^18^FDG-PET CT, 46 had had serous cavity effusions, often affecting more than one cavity [17]. The four most common sites were pleural, pelvic, pericardial and abdominal.

#### Endocrinopathy

In a retrospective study of 64 patients with POEMS syndrome, 84% had endocrinopathy, 54% of whom had multiple endocrinopathies [20]. Hypogonadism was commonest, affecting 79% of men. Other endocrine abnormalities described include thyroid dysfunction, abnormal calcium metabolism, glucose intolerance, diabetes, hyperprolactinemia, gynaecomastia, and less commonly adrenal insufficiency [20–22]. For diagnostic purposes, diabetes and thyroid disease alone are insufficient evidence of endocrinopathy, given the high incidence of both conditions separately in the general adult population [1].

#### Skin changes

Skin changes are reported in as many as 90–100% of patients [6, 23, 24]. In a study of 107 patients with POEMS syndrome, patients had an average of 3 types of skin changes [23]. Hyperpigmentation and haemangiomas are the commonest findings [23, 24]. Other skin changes include skin thickening, hypertrichosis, acquired facial lipoatrophy, and infiltrated livedo reticularis with necrosis [23, 24]. Vascular-type skin changes can include acrocyanosis, flushing, rubor, hyperaemia and Raynaud’s phenomenon [23, 24]. Nail changes include leukonychia and clubbing. Skin ulceration related to calciphylaxis can occur [25]. There is a significant association between the presence of skin changes and abnormal pulmonary function tests, suggesting these patients be meticulously screened for respiratory complications [23].

#### Papilloedema

Optic disc swelling is often visible on bedside direct ophthalmoscopy, with detection aided by formal ophthalmology assessment and optical coherence tomography (OCT). In a retrospective study of 33 patients, 52% had bilateral disc swelling [26]. In some patients with disc swelling, raised CSF opening pressures have been described, though this is unlikely to be the only causative factor [26]. Elevated CSF protein, often linked to optic disc swelling in other inflammatory neuropathies, is present in POEMS syndrome [6]. Increased vascular permeability secondary to elevated VEGF may also be important. In a study of 17 patients with POEMS syndrome, serum VEGF concentrations were significantly higher in patients with disc swelling compared to those without, and there was a positive correlation between serum VEGF levels and peripapillary retinal thickness on OCT [27]. None of the studied patients had elevated CSF opening pressures. Improvement of disc swelling has been shown to correlate with fall in VEGF concentrations [26].

#### Thrombocytosis and Polycythaemia

Thrombcytosis and polycythaemia commonly occur, however, testing for JAK2 mutation is typically negative [12].

### Other described associations

#### Pulmonary symptoms

Pulmonary hypertension (PH) was found in 27% of a cohort of 154 patients with POEMS syndrome [28]. Unlike with primary PH, PH in POEMS syndrome tends to follow a less progressive, less severe course, and improve with treatment [28, 29]. In a different cohort, 28% of 137 patients had chest symptoms within 2 years of diagnosis [30]. The presence of respiratory muscle weakness is associated with a poorer survival outcome [30].

#### CNS involvement

In a study of 11 patients with POEMS syndrome, 9 had asymptomatic cranial pachymeningeal thickening on gadolinium-enhanced MRI [31]. Meningeal biopsy in two patients showed meningothelial cell proliferation with vascular changes including neovascularisation, and narrowing/occlusion of arterioles due to thickening of the media and endothelial hyperplasia. The role of VEGF was suggested by immunohistochemical staining for VEGF and VEGFR2.

#### Arterial vascular disease

Cardio- and cerebrovascular risk may be increased in POEMS syndrome. In a cohort of 90 patients, the estimated 5-year ischaemic stroke risk was 13.4% [32]. Risk factors included degree of thrombocytosis on presentation and presence of bone marrow plasmacytosis. There were no cases of stroke in patients with treated POEMS syndrome and the authors suggested that treating the underlying syndrome was probably the best mechanism for reducing arterial risk.

#### Renal disease

Renal dysfunction has been documented to varying degrees. In a retrospective study of 299 patients with POEMS syndrome from China, 67 had renal impairment, mostly of moderate degree [33]. Microhaematuria and significant proteinuria were present in 29 and 17 patients, respectively. In other cohorts, the incidence of renal dysfunction is lower [1]. In the Chinese cohort, renal function improved with treatment in 66%. Early death and reduced overall survival were significantly likelier in patients who had severe renal dysfunction (estimated glomerular filtration rate < 30 ml/min/1.73 m^2^) at baseline. Pathological findings on renal biopsy, presumably secondary to the effects of VEGF and pro-inflammatory cytokines, include glomerular enlargement, with endothelial and mesangial cell proliferation and small artery vasculopathy [33, 34].

## Patient evaluation and investigations

When POEMS syndrome is suspected, a detailed history and systems examination are essential. Recommended investigations are listed in Table 2. CSF examination usually reveals an elevated protein in the range of 1–2 g/L but a normal cell count [6].

**Table 2:** Useful investigations

Laboratory studies
Full blood count
Clotting profile
Renal and liver function tests
Endocrinopathy screen
Paraprotein screen inc. serum protein electrophoresis, immunofixation, serum free light chains (+ urine Bence Jones proteins)
VEGF level
Cerebrospinal fluid analysis
Imaging
^99m^Tc-HMDP bone scintigraphy
FDG-PET CT
Histology
Iliac crest bone marrow aspirate/biopsy
± lymph node biopsy
Nerve biopsy (if haematological studies inconclusive)

### Differential diagnosis

#### Chronic inflammatory demyelinating polyradiculoneuropathy

CIDP is an inflammatory, proximal and distal sensory-motor neuropathy, with demyelinating features. Differences between CIDP and POEMS syndrome are listed in Table 3 [3]. Unlike POEMS syndrome, CIDP usually responds well to monotherapy with intravenous immunoglobulin (IVIg) or steroids [35].

**Table 3:** **POEMS vs CIDP** [3, 8, 36, 37]

	POEMS	CIDP
Clinical findings	Distal and proximal sensory and motor neuropathy	Distal and proximal sensory and motor neuropathy
	Often painful	Painless
Neurophysiology	Demyelinating polyradiculoneuropathy with axonal loss	Demyelinating polyradiculoneuropathy
Distal motor latency	Prolonged	More prolonged than POEMS
Conduction velocities	Decreased (demyelinating range)	Similarly decreased (demyelinating range)
Compound muscle action potentials	Significantly attenuated, often absent	Less severely attenuated
Lower limb vs upper limb discrepancy	Significantly worse in lower limbs compared to upper limbs	Less discrepancy between upper and lower limbs
Distribution	Intermediate nerve segments most affected	Proximal and terminal nerve segments most affected
Neuropathology	More severe axonal loss	
	Diffuse loss of myelinated fibres	Multifocal loss of myelinated fibres
	Degree of epineurial inflammation	Endoneurial mononuclear cell infiltration and degree of epineurial inflammation
	Epineurial vascular proliferation	
	Uncompacted myelin lamellae > 1%	Onion bulb formation
Biomarkers	Elevated VEGF	Antibodies to paranodal proteins and gangliosides in a minority of cases

#### Monoclonal gammopathy of undetermined significance (MGUS)

MGUS is a pre-malignant plasma cell disorder, with IgM, non-IgM or light chain subgroups, which can transform into Waldenstrom’s macroglobulinaemia (WM), myeloma or AL amyloidosis [38]. There is a male predominance, and age of onset is typically higher than for POEMS syndrome [9]. Neuropathy is not required for diagnosis. Unlike POEMS syndrome, neuropathy, when present, is mostly associated with IgM paraproteins, usually IgM kappa [9] and many patients have antibodies targeting myelin-associated glycoprotein (MAG). The neuropathy in these patients is clinically different from POEMS syndrome: typically sensory and painless, often with sensory ataxia and tremor [39]. Progression is slow, and neuropathy can be mild for many years. A watch-and-wait approach is commonly adopted, with decision to treat based on severity and rate of progression.

#### Waldenstroms macroglobulinaemia (WM)

WM is a malignant plasma cell dyscrasia, with an IgM paraprotein and > 10% infiltration of lymphoplasmacytic cells in the bone marrow [40]. It is usually indolent but can transform into a high-grade lymphoma. Like MGUS, there is a male predominance, and age of onset is usually older than POEMS syndrome. Around half of patients may have a symptomatic neuropathy, typically a distal sensory neuropathy, clinically similar to MGUS, but with more axonal features on electrodiagnostic testing [41, 42]. Systemic symptoms can occur due to very high paraprotein levels and associated hyperviscosity. Systemic signs in WM can include hepatosplenomegaly, lymphadenopathy and anaemia. Treatment involves a rituximab-based chemotherapy regime, monitoring for hyperviscosity, and supportive treatment [40, 43].

#### Myeloma

Myeloma is a malignant plasma cell disorder of bone marrow, causing osteolytic bone lesions and end-organ damage including anaemia, hypercalcaemia and renal failure [44]. The percentage of malignant plasma cells in bone marrow is much higher than in POEMS syndrome. Neuropathy attributable to disease is less common in myeloma than MGUS and WM and may often be attributable to chemotherapy rather than the disease process. Neuropathy is typically axonal but more heterogeneous than in MGUS, WM or POEMS syndrome. Treatment strategies target the plasma cell clone [44]. Most of the treatments for POEMS syndrome were first used in myeloma.

#### Immunoglobulin light chain (AL) amyloidosis

AL amyloidosis is an acquired disorder caused by monoclonal, non-malignant plasma cells producing immunoglobulin light chains, most often lambda, which misfold into insoluble beta-pleated sheets and deposit in the tissues [45]. Histopathological identification of amyloid is achieved using Congo red staining with apple-green birefringence under polarised light. Like POEMS syndrome, AL amyloidosis has a number of characteristic systemic complications, though these are secondary to AL amyloid deposition. The complications include cardiomyopathy, hepatomegaly, nephrotic syndrome, fatigue and weight loss. Around a quarter of patients have polyneuropathy, which is typically painful like in POEMS [46]. In contrast, however, amyloid neuropathies are usually axonal and feature prominent autonomic involvement [46]. Amyloid is characteristically absent in nerve and bone marrow biopsies in patients with POEMS syndrome [6]. The prognosis of AL amyloidosis without treatment is poor, especially in patients with cardiac involvement [45]. Treatment options are similar to POEMS syndrome, and include autologous stem cell transplant (ASCT) or chemotherapy [45, 47].

#### CANOMAD

Chronic Ataxic Neuropathy with Ophthalmoplegia, M-protein, Cold Agglutinins and Disialosyl antibodies (CANOMAD), is an antibody-associated neuropathy, caused by IgM antibodies targeting disialylated and polysialylated gangliosides, including GD1b, GD3, GT1b and GQ1b [48]. Light chains may be kappa or lambda restricted or both [48]. The origin of the IgM antibodies is unclear; only some cases are reported to have an underlying plasma cell dyscrasia. Clonal B-cell infiltration in cranial and peripheral nerves and nerve roots has been described in one case [49]. Similar to POEMS syndrome, there is a male predominance and age of onset is typically in the 6th decade [48]. The neuropathy is often severe, but tends to be more slowly progressive than in POEMS syndrome, and predominantly sensory, often with a disabling sensory ataxia, and either demyelinating or axonal features on electrodiagnostic testing [48, 50].

## Management

Evidence for treatment in POEMS syndrome is largely limited to retrospective cohort studies, with only one randomised controlled trial (RCT) to date [51, 52]. IVIg or steroid monotherapy, commonly used in other inflammatory neuropathies, does not produce lasting benefit.

The current suggested treatment algorithm recommends localised radiotherapy for patients with localised disease, defined as up to 3 discrete bone lesions and no evidence of clonal plasma cells on iliac crest biopsy, or systemic treatment in patients with diffuse disease, defined as > 3 bone lesions or clonal plasma cells on iliac crest biopsy [53]. Systemic treatment options include ASCT or chemotherapy. Supportive treatment for neurological disability and systemic symptoms should also be considered [52, 53].

### Radiotherapy

In a retrospective study of 146 patients treated at the Mayo clinic, 38 patients received radiotherapy as a first definitive treatment for POEMS syndrome [54]. 54/66 of identified bone lesions were irradiated. Overall survival was 97% at 4 years, including patients requiring salvage therapy, suggesting that even in patients where radiotherapy fails to prevent progression, trialling it first line may not negatively affect survival, and may prevent unnecessary systemic therapy in responders. Response to treatment was mixed: event free survival was 52% at 4 years, 47% had clinical improvement, and 48% required salvage treatment for poor response, progression or relapse. Patients with better haematological responses had lower risk of treatment failure. A retrospective study of 33 patients, treated in Seoul, also showed that radiotherapy was effective in improving clinical symptoms of POEMS syndrome in some patients, either alone or in combination with chemotherapy [55].

### Alkylating chemotherapy agents

Melphalan can be effective with or without ASCT. In a single centre prospective study of 31 patients using a 12-cycle treatment regime of melphalan and dexamethasone, complete and partial haematological responses were achieved in 38.7% and 41.9% patients, respectively [56]. All had improvement in neurological symptoms. There were no treatment-related deaths. It was suggested that melphalan with dexamethasone was safe and effective, without the cost and treatment-related morbidity of ASCT, but longer term conclusions were limited by the short follow-up period (median 21 months).

### Autologous stem cell transplant

Two large retrospective cohort studies support the use of ASCT in POEMS syndrome [57, 58]. Further studies are needed to compare relative risks and benefits of ASCT to other systemic treatments [57]. Though modifications to the process of ASCT have been made, significant risks remain, including engraftment syndrome, chemotherapy-associated risks, graft failure, pancytopaenia, serious infection, and death.

The largest cohorts receiving ASCT, from Europe (127 patients) and the Mayo clinic (59 patients), reported a similar 5-year progression free survival of 74% and 75%, with patients followed up for a median 48 months and 45 months, respectively [57, 58]. Early responses included improvement in VEGF levels and systemic features such as volume overload [57]. In patients who progressed in the Mayo group, median time to progression was 43 months. Most relapses were radiological or VEGF-related, without clinical symptoms. Patients who progressed were still felt to have benefitted from ASCT, and most responded well to second line therapy or observation. There were 13 and 3 deaths in the European and Mayo cohorts, respectively.

Neurological symptoms have been shown to improve post-ASCT. In a study of 60 patients followed up over a median 61 months, wheelchair use improved from 45 to 0% post-transplant and there was a reduction in the number of patients requiring walking aids and ankle–foot orthoses [59].

### Thalidomide and lenalidomide

In the first RCT in POEMS syndrome, 25 patients eligible for systemic therapy but not ASCT, were randomised to either thalidomide + dexamethasone or placebo + dexamethasone for 24 weeks, before proceeding to a 48-month open-label safety study where all patients received thalidomide [51]. At 24-weeks, reduction rate of VEGF (the primary endpoint) was significantly greater in the treatment group. Motor outcome and two SF-36 quality of life scores were also significantly improved in the treatment groups, but other secondary outcome measures were not. A new sensory neuropathy was found in 23% of patients in the open-label group, likely thalidomide-related, however, overall nerve function, particularly motor function, improved. Mild bradycardia was common. Overall, despite the short trial period, it was concluded that thalidomide was a safe and effective treatment for POEMS syndrome.

Lenalidomide is structurally similar to thalidomide, but less neurotoxic. Two recent prospective studies, a number of small retrospective studies and a pooled analysis study have all shown it to be effective in POEMS syndrome, as either first- or second-line therapy [60–65].

### Bortezomib

Bortezomib (Velcade) is a reversible proteosome inhibitor and approved by NICE for use with dexamathasone ± thalidomide in myeloma and untreated mantle cell lymphoma. He et al. studied 20 patients, newly diagnosed with POEMS syndrome between Dec 2014 and May 2016, given 3–6 cycles of induction therapy with reduced dose bortezomib, cyclophosphamide and dexamethasone [66]. Of those whose haematological response could be assessed, 7 showed complete response, 6 partial response and 4 had stable disease. Despite concerns about bortezomib causing peripheral neuropathy, no patient had worsening neurology and 19/20 had improvement in ONLS (Overall Neuropathy Limitations Scale) of at least 1 point. Systemic features also improved. No deaths or disease progression were seen during the follow-up period, though limited to 11 months.

### Therapies targeting VEGF

Bevacizumab is a monoclonal antibody targeting VEGF. A number of small studies and case reports have shown mixed results with bevacizumab, including treatment failure (except VEGF response), and worsening of disease leading to death [67–70]. Some case studies have shown benefit, though these have been limited by short or unclear follow-up periods, and bevacizumab has been used following, or in addition to other treatments including cyclophosphamide with dexamethasone [68, 71]. A small number of studies have suggested that short-term use of bevacizumab may be helpful as a bridge to definitive treatment such as ASCT, either alone or with other treatment such as thalidomide [72, 73]. At this stage, the role of bevacizumab is unclear, and better understanding of the pathophysiology of POEMS syndrome is required before it can be recommended.

## Follow-up and outcomes

Post-treatment, prognosis in POEMS syndrome is good; in a study of 291 patients treated at the Mayo clinic between 1974 and 2014, 10-year overall survival was 62%, with younger age, albumin > 3.2 g/dL and complete haematological response to treatment all associated with better outcomes [74]. In an overlapping cohort, 5-year progression-free survival was 58%, with failure to achieve complete haematological response after first-line treatment the main predictor of relapse or progression [75]. Even in patients who relapsed, however, prognosis was good: 92% of patients responded to second line treatment, and most who relapsed again responded to further treatment. Given the risk of relapse, close follow-up of clinical, radiological and biochemical parameters, is essential.

## Conclusions

POEMS syndrome is a rare, but treatable cause of neuropathy. Further work is required to establish its exact underlying pathophysiology. Current treatment approaches afford good prognosis. Moving forward, randomised-controlled studies, though difficult given the rarity of POEMS syndrome, and the development of prognostic tools, will be important in establishing individualised approaches to patient care.
